# Are Breaks in Daily Self-Weighing Associated with Weight Gain?

**DOI:** 10.1371/journal.pone.0113164

**Published:** 2014-11-14

**Authors:** Elina E. Helander, Anna-Leena Vuorinen, Brian Wansink, Ilkka K. J. Korhonen

**Affiliations:** 1 Department of Signal Processing, Tampere University of Technology, Tampere, Finland; 2 VTT Technical Research Centre of Finland, Tampere, Finland; 3 Charles S. Dyson School of Applied Economics and Management, Cornell University, Ithaca, New York, United States of America; University of Alabama at Birmingham, United States of America

## Abstract

Regular self-weighing is linked to successful weight loss and maintenance. However, an individual's self-weighing frequency typically varies over time. This study examined temporal associations between time differences of consecutive weight measurements and the corresponding weight changes by analysing longitudinal self-weighing data, including 2,838 weight observations from 40 individuals attending a health-promoting programme. The relationship between temporal weighing frequency and corresponding weight change was studied primarily using a linear mixed effects model. Weight change between consecutive weight measurements was associated with the corresponding time difference (*β* = 0.021% per day, *p*<0.001). Weight loss took place during periods of daily self-weighing, whereas breaks longer than one month posed a risk of weight gain. The findings emphasize that missing data in weight management studies with a weight-monitoring component may be associated with non-adherence to the weight loss programme and an early sign of weight gain.

## Introduction

Regular self-monitoring of body weight is an effective intervention in weight loss programmes and, especially, in weight maintenance [1]–[3]. Frequent self-monitoring is assumed to improve self-awareness, provide early detection of subtle weight increases and prevent weight regain after weight loss [2]. Regular self-weighing is recommended as part of the behavioural therapy for weight management by the National Institute of Health and is deemed ‘crucial’ for long-term weight maintenance [4]. Frequent self-monitoring may also promote weight management during the holiday season when the risk of weight gain is high [5].

Van Wormer et al. [6] reviewed 12 studies that used self-weighing as an intervention for weight loss and weight maintenance. In 11 of the studies, more frequent self-weighing was associated with greater weight loss or weight gain prevention: weekly and daily self-weighers held a 1–3 BMI unit advantage compared with individuals who did not weigh themselves as frequently. For a 1.7 m-tall person this means a 3–9 kg lower weight. In the study by Linde et al. [7], daily self-weighing was associated with the greatest weight loss outcomes compared with self-weighing on a weekly, monthly or semi-monthly basis, or never. Van Wormer et al. [3] used self-weighing frequency to predict a weight change over two years; more frequent self-weighing was associated with slower weight (re)gain and, for obese individuals, also possibly weight loss. Fujimoto et al. [8] found that subjects who were instructed to weigh themselves as frequently as four times a day and draw a graph of their weight lost twice as much weight as the group that only had behaviour therapy. In a 20-week study by Gokee-LaRose et al. [9], one group was instructed to obtain daily weights with a digital memory scale whereas the other group was instructed not to weight themselves until week 11 and then to obtain weekly weights. Both groups received behavioural therapy. There was no significant difference in weight loss between the groups, but weighing frequency and weight loss were associated. Though it seems that frequent self-weighing is advantageous, the optimal self-weighing frequency is not known [3].

Studies that investigate the effect of self-weighing frequency on weight control do not usually analyse self-weighing data, but evaluation is often based on a retrospectively self-reported monitoring frequency. Participants are typically asked to summarize their long-term self-weighing as being on a daily, weekly or monthly basis [1], [3], [7]. However, categorized response options may not reflect true weighing behaviour over time [10], added to the fact that the self-weighing frequency may vary greatly over time. Both Van Wormer et al. [10] and Gokee-LaRose et al. [9] studied the actual self-weighing frequency using a weight scale that automatically transmitted or saved weight information to counsellors. An association between frequent self-weighing and greater weight loss was found in both studies. However, in these studies, the self-weighing information was reduced to a single frequency value that does not reflect temporal variations in self-weighing activity.

Weight management is a life-long task for an individual. Detecting risky periods when weight might start to increase would be important in preventing significant weight gain. This paper examines the association between temporary adherence and non-adherence to daily self-weighing and temporary weight changes using self-weighing data from individuals who participated in a health promotion programme. Our hypothesis is that breaks in self-weighing are temporally associated with an increased risk of weight gain.

## Methods

### Weight Data

Self-recorded weight measurements were aggregated from an earlier study [11], [12]. The study was approved by the Ethics Committee of Helsinki and Uusimaa Hospital District, Finland. Written informed consent was obtained from all participants. The procedure was approved by the ethics committee. The consent was documented by the principal investigator at the Finnish Institute of Occupational Health and stored in a secured locker that could only be accessed by appointed researchers. The study was part of a workplace health promotion programme in Finland during 2008–2009, and all the participants were white Caucasians. The health promotion programme (eight-week intervention period with a one year follow-up period) was intended to improve the participants' health risk profile by promoting weight loss, fitness, a healthy diet, cessation of smoking, stress reduction, and a decrease in alcohol intake. The participants were not paid.

Prior to the intervention, the participants were asked if they intended to lose weight and to name the health issue they found most challenging or the one that required most improvement. Participants were encouraged to self-monitor those variables they sought to improve on a daily basis. Participants seeking to lose weight were instructed to self-monitor and record their daily weight immediately after waking up and before breakfast with a mobile phone application [12]. Participants were provided with weight scales. The weighing data were extracted from the mobile phone application at the one-year follow-up. Details of the study can be found elsewhere [13].

For the 117 individuals participating in the health promotion study, we set four inclusion criteria to obtain meaningful data for this post hoc analysis. The inclusion criteria were a) having a body mass index (BMI) of at least 25 kg/m^2^ and b) having a weight loss target (identified from a questionnaire as either intending to lose weight and/or considering weight to be the health issue that most challenged them or would require most improvement). Participants without a weight loss target or need for it (normal weight or underweight) were hence not included. For eligible participants, the inclusion criteria for self-weighing data were having at least five weight measurements and a minimum self-weighing period of 30 days. The duration of the self-weighing period was determined from the time the subject made his/her first entry to the time the subject made his/her last entry.

Forty participants fulfilled the inclusion criteria. Table 1 describes the population in the original study and the sample chosen for this study with statistics of the self-weighing period for the included subjects. The total number of included weight measurements was 2838.

**Table 1:** Inclusion criteria and characteristics of the original study population and included individuals (mean±standard deviation(range)).

	Original study population	Included individuals
**Number of individuals**	**117**	**40**
**Sex [number of males] (percentage)**	**35 (30%)**	**13 (33%)**
**Age [years]**	**44** a **±7** b **(30–55)** c	**45±6 (33–54)**
**BMI [kg/m^2^]**	**28±4 (20–41)**	**29±3 (25–34)**
**Duration of self-monitoring [days]**		**247±111 (39–391)**
**Number of measurements per individual**		**71±85 (5–330)**
**Total number of weight measurements**	**3455**	**2838**

a mean,

b standard deviation,

c range.

The differences in the number of males and age between the original study population and the included sample population are not significant (all *p*>0.43). The difference in BMI is significant (*p* = 0.030) due to the inclusion criterion of having a BMI of at least 25.

### Data analysis

All analytical procedures were performed using MATLAB version 2013b (The MathWorks Inc.). Daily weight measurements were normalized by dividing them by each individual's average weight (% changes). For each individual, the weight change between two consecutive measurements (%) and the corresponding time differences in days were calculated. Negative and positive weight differences indicate weight loss and weight gain, respectively. In total, there were 2798 weight change observations.

The primary analysis of the study involved examining the dose-response relation between the frequency of self-weighing and the corresponding weight change. The analysis was done using linear mixed effects modelling (procedure *fitlme* in MATLAB with the maximum likelihood estimation method) with weight change as the response variable and the day difference between consecutive weight measurements entered into the model as fixed effects. The model was also adjusted for baseline weight. Random effects accounted for individual-level variation, and they were uncorrelated. The aim was to examine whether there is a significant trend, a linear coefficient *β*, in weight change as a function of days between weight measurements. Furthermore, the presence of a significant constant term α denotes that there is weight change different from zero between measurements taken on consecutive days (i.e. daily self-monitoring).

In the secondary analysis, self-monitoring frequency (based on the time difference between consecutive weight measurements) was divided into four categories that are generally used in self-weighing studies to characterize monitoring frequency. The categories determined were

1. No break between measurements (‘Daily’)
2. 1–6 days without measurements, consecutive measurements taken at least weekly (‘At least weekly’)
3. 7–29 days without measurements, consecutive measurements taken at least monthly (‘At least monthly’)
4. 30 days without measurements, consecutive measurements taken less than monthly (‘Less than monthly’)

An individual's observations may fall into multiple categories, but the analysis in this part is based on weight difference observations that were not clustered at individual level. The student's t-test was used to test if the weight changes differed from zero, denoting significant weight change.

The level of significance in all tests was 0.05.

## Results

**Figure 1:**
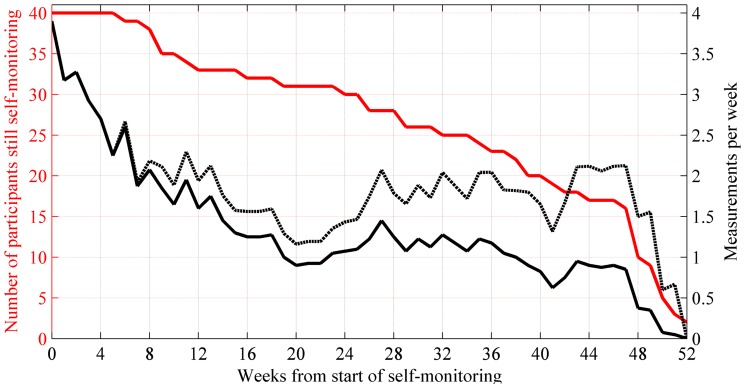
Self-weighing patterns in time as a number of subjects involved in weight-monitoring and the number of weekly weight measurements. The left y axis (red) corresponds to the number of participants that is still involved in self-monitoring and the red line shows the participant numbers for each week since starting the self-monitoring. The right y axis (black) corresponds to the number of measurements per week. Black dashed and solid lines show the weekly self-monitoring frequency average from two subgroups: subjects that are still actively self-monitoring (dashed line) and all study subjects (solid line).

Figure 1 illustrates self-weighing patterns in time by showing the number of subjects still carrying out weight monitoring and the average number of weight measurements on a weekly basis over the monitoring weeks. Two people continued self-monitoring after 52 weeks, which is not shown in the figure. The adherence to frequent self-weighing decreases over time.

Self-weighing frequency was inversely related to weight change. Weight loss was significantly decelerated or weight increased when the day difference between two consecutive weight measurements became longer (Table 2). Controlling for baseline weight did not alter the results (*β* = 0.0207 for day difference, *p*<0.001). Random effects were uncorrelated (*r*(38) = 0.12, *p* = 0.47). The significant intercept indicates that when the day difference between two measurements was zero (i.e. daily self-weighing), the weight change was negative. The theoretical minimum self-weighing interval (days without measurements) with no weight gain was obtained by solving y = 0 (no weight change) from the regression equation. This was equal to 5.8 days, which corresponds approximately to weekly self-weighing.

**Table 2:** Linear mixed effects model summary table for predicting weight change as a function of days between consecutive weight measurements.

Fixed effects	Parameter estimate (95% CI)	t-value	p-value
**Intercept**	**−0.121 (−0.167, −0.075)**	**−5.16**	**<0.001**
**Day difference**	**0.021 (0.011, 0.030)**	**4.28**	**<0.001**
**Random effects covariance**	**STD (95% CI)**		
**Intercept**	**0.082 (0.039, 0.174)**		
**Day difference**	**0.025 (0.017, 0.036)**		
**Residual**	**0.764 (0.744, 0.785)**		

**CI = confidence interval, STD = standard deviation.**

**Figure 2:**
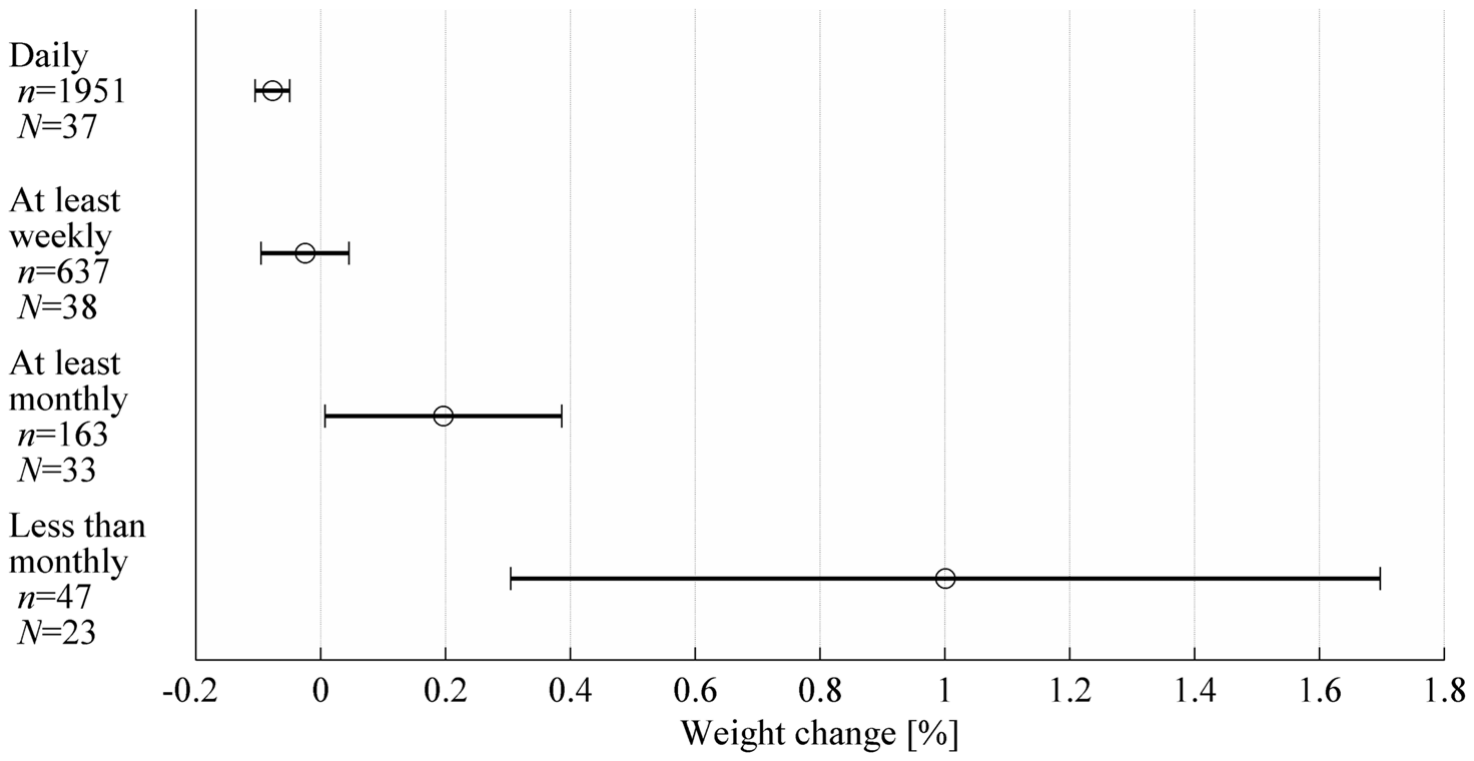
Mean weight change (circle) and the 95% confidence intervals (horizontal lines) in different self-weighing categories. The data were gathered from 40 subjects: *n* denotes the number of observations in each category and *N* denotes for the number of subjects that had at least one observation in a category.

**Figure 3:**
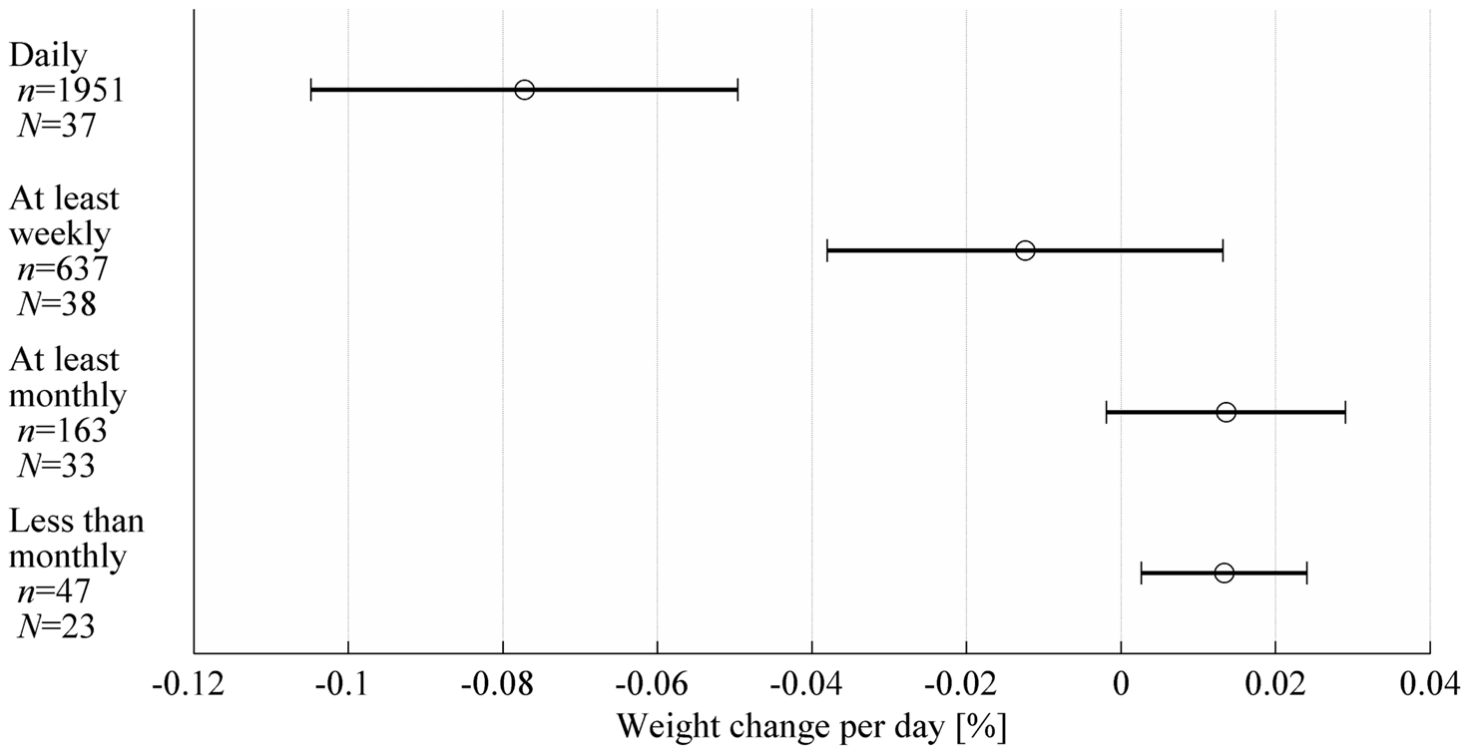
Mean weight change per day (circle) and the 95% confidence intervals (horizontal lines) in different self-weighing categories. The data were gathered from 40 subjects: *n* denotes the number observations in each category and *N* denotes for the number of subjects that had at least one observation in a category.

The secondary analysis shows that during periods of daily weighing, the subjects generally lost weight (Figure 2, ‘Daily’ category, *t*(1950) = −5.48, *p*<0.001) whereas weight gain was associated with breaks longer than a week (Figure 2, ‘At least monthly’ category, *t*(162) = 2.05, *p* = 0.042) or a month (Figure 2, ‘Less than monthly’ category, *t*(46) = 2.89, *p* = 0.006). Weight change did not significantly differ from zero when self-weighing was carried out at least weekly (Figure 2, ‘At least weekly’ category, *t*(636) = −0.70, *p* = 0.49). The average number of days between consecutive weight measurements was 2.4 in the ‘At least weekly’ category, 13 in the ‘At least monthly’ category and 72 in the ‘Less than monthly’ category. Figure 3 shows weight change per day in the corresponding categories. The level of significance for weight change per day differing from zero was *p*<0.001 (*t*(1950) = −5.48) for ‘Daily’, *p* = 0.34 (*t*(636) = −0.95) for ‘At least weekly’, *p* = 0.08 (*t*(162) = 1.74) for ‘At least monthly’, and *p* = 0.016 (*t*(46) = 2.50) for ‘Less than monthly’. The results were similar whether cumulative weight changes or weight change per day were analysed except for ‘At least monthly’ category in which weight increase per day was not significant.

## Discussion

This study demonstrates a positive relationship between frequent self-weighing and successful weight control by analysing temporal associations between time difference of consecutive weight measurements and the corresponding weight changes based on actual self-weighing data. We found a significant dose-response relation between self-weighing frequency and weight change. Weight increased linearly with the number of days between consecutive weight measurements: the more days between weight measurements, the larger the weight gain. The results were consistent whether subject-level analysis (mixed effects model) or observation-level analysis was used. The analyses were based on data inherited from overweight individuals who intended to lose weight or considered weight to be the health issue that most challenged them or would require most improvement and who were participating in a health promotion programme that encouraged daily self-monitoring of weight.

The results of the secondary analysis showed that daily self-weighing was generally associated with weight loss while breaks longer than one week and, especially, longer than one month were associated with weight gain. The findings were consistent whether cumulative changes or weight changes per day were analysed. The primary analysis showed that the theoretical minimum self-weighing interval associated with unchanged weight was 5.8 days, indicating approximately weekly self-weighing. Correspondingly, in the ‘At least weekly’ category, the weight change did not differ from zero and weight measurements were taken on average 2–3 times a week. As discussed by Phelan et al. [14], during weight loss maintenance, it can be difficult to reverse the trajectory of even minor weight gains. Therefore, individuals seeking weight maintenance may be encouraged to conduct self-weighing at least weekly. We note that categorized self-weighing frequencies were not data-driven but based on general understanding to make results comparable with other studies in the field.

Our findings were consistent with studies such as [2], [3]. Interestingly, Linde et al. [7] found that in a weight gain prevention trial, a negative BMI change was associated with daily weighing, whereas in a weight loss trial, weekly or daily self-weighing was associated with a decrease in BMI. In the randomized controlled trial (RCT) by Gokee et al. [9], which directly manipulated the weighing frequency between the two groups (daily weighing vs no weighing during the first weeks, then weekly weighing) and in which the groups received similar behavioural therapy as an intervention, the researchers found no difference in weight loss between the groups. In an RCT by Wing et al. [15], the researchers studied the effect of pre-specified breaks (2-week and 6-week breaks during which participants were advised to stop all weight loss attempts) on weight control. During the breaks, participants weighed themselves 2–3 times a week whereas at other times, self-weighing was done approximately 5 times a week. The researchers found that the breaks did not alter the long-term weight change and that all the groups had lost comparable amount of weight during the 14-week programme. However, the breaks produced slowing of weight loss or slight weight regain. These findings on short-term weight change are consistent with the findings of our study in which weight change did not differ from zero if self-weighing was done on a weekly basis, 2–3 times a week on average.

The benefits of frequent self-weighing are not unambiguous however. For some individuals, frequent self-weighing may be associated with negative outcomes such as increased body dissatisfaction or decreased self-esteem when progressing too slowly towards or failing in weight loss goals [16]. Nevertheless, self-weighing frequency and body satisfaction were not found to correlate in a weight loss trial, whereas increased self-weighing frequency was once again associated with greater weight loss [17].

A major strength of this study was the use of actual and varying self-weighing data. By using these data, it was possible to analyse the temporal association between self-weighing and weight change instead of evaluating the relationship retrospectively. Another advantage is that the results were based on longitudinal data. By analysing repeated weight observations in individuals, the measurement bias does not play such a big role. Weight is known to vary over the time of the day, day of the week [18], month and holiday season [19]. Repeated measurements obtained from the same individual smoothens this variation. The average follow-up time was 247 days with 71 weight entries.

Limitations include the small sample size and homogenous population. The sample represented a rather selected group of individuals who were employed and overweight (BMI>25), had signed up for a health promotion programme and were motivated to achieve weight loss. Many weight loss studies using self-monitoring suffer from the problem of sample homogeneity, namely the predominance of white women [20]. Secondly, the data do not provide evidence on whether changes in weight or changes in self-weighing frequency occurred first. It may be speculated that the reported association is related to adherence to the overall intervention: a lack of daily self-weighing in this group may be a sign of non-adherence to weight management. We cannot conclude that daily self-monitoring of weight leads to weight loss, but regular self-weighing may lower the risk of gaining weight in overweight or slightly obese subjects. Thirdly, there was a significant drop-out rate in weight self-monitoring during the follow-up. Only half of the subjects weighed themselves at least once after 40 weeks, and low long-term adherence to self-monitoring reduces its wide applicability and calls for methods to reduce long-term attrition. This study served as a preliminary investigation and the results should be confirmed in larger studies with randomized controlled settings. Randomized trials such as [9], [15] have evaluated the benefits of self-weighing frequency in a controlled setting but neither evaluated the efficacy of self-weighing frequency alone, without a behavioural therapy component. Based on our results, self-monitoring of weight may be useful to do more than once a week, and its efficacy is worth further examination. It is also notable that the efficacy of self-weighing may be dependent on the target population. As found by Linde et al. [7], weight loss maintainers and weight losers benefit from different self-weighing frequencies. Benefits of self-weighing frequency are not studied in the context of weight loss maintenance or weight maintenance to our knowledge.

There is an increasing number of weight scales available that automatically save weights to a web server. Thus, in the future, individuals may have weight data from long monitoring periods such as multiple years. A detailed analysis of weight changes during active and less active monitoring periods, similar to this study, could be provided to people to find their optimal individual weighing frequency. Having a self-weighing frequency adapted to an individual's data supports the idea of individualizing self-monitoring strategies [21].

## Conclusions

The frequency of self-weighing and weight changes were temporally associated in participants who were overweight (BMI>25), had a weight loss target and were advised to self-monitor on a daily basis. Long breaks in self-weighing were associated with a risk of weight gain, whereas weight loss typically took place during active (i.e. daily) self-weighing periods. The findings emphasize that missing data in weight management studies with a weight-monitoring component may be associated with non-adherence to the weight loss programme and may be an early sign of weight gain.
